# A screening pipeline to characterize stress-induced enzymes uncovers a cellular function for the poorly characterized alcohol dehydrogenase Bdh2

**DOI:** 10.1242/jcs.264913

**Published:** 2026-06-12

**Authors:** Dunya Edilbi, Rosario Valenti, Benjamin Dubreuil, Yeynit Asraf, Yoav Peleg, Shira Albeck, Sergey Malitsky, Maxim Itkin, Maya Schuldiner

**Affiliations:** ^1^ Department of Molecular Genetics, Weizmann Institute of Science, Rehovot 7610001, Israel; ^2^ Structural Proteomics Unit (SPU), Life Sciences Core Facilities (LSCF), Weizmann Institute of Science, Rehovot 7610001, Israel; ^3^ Metabolic Profiling Unit (MPU), Life Sciences Core Facilities (LSCF), Weizmann Institute of Science, Rehovot 7610001, Israel

**Keywords:** Enzymes, Stress conditions, Cell metabolism, Metabolites, Metabolomics, Alcohol dehydrogenase

## Abstract

Cells possess intricate metabolic networks comprising hundreds of enzymes. Despite extensive research, many of these enzymes remain uncharacterized. Identifying the function of these enzymes is crucial for advancing our understanding of cellular metabolism. However, multiple enzymes are not active in standard conditions, making them challenging to study. To overcome this challenge, we created a pipeline to track the upregulation of enzymes at the protein level during diverse growth conditions, suggesting a requirement for their activity in these conditions. To do this, we assembled a collection of ∼180 yeast strains, each containing an uncharacterized putative enzyme fused to a fluorophore and under the regulation of its own promoter. By subjecting the collection to 42 diverse environments, we identified the biologically relevant conditions for the upregulation of 16 proteins. We focused on one such putative alcohol dehydrogenase, Bdh2, whose expression was upregulated during nutrient-limited conditions, and functionally characterized it. More broadly, our discovery pipeline lays the foundation for uncovering new stress-induced enzymes. This has implications for the cell biology of metabolism and biotechnology.

## Teaser

**Summary:** A stress-responsive screening pipeline identifies condition-induced enzymes and reveals that the poorly characterized alcohol dehydrogenase Bdh2 promotes metabolic adaptation during nutrient limitation.

## INTRODUCTION

Understanding the intricate workings of the cell necessitates a comprehensive knowledge of all its components. Cellular metabolism consists of the regulated biochemical processes carried out by enzymes organized in metabolic pathways. To preserve homeostasis, a cell adjusts its specific needs by carefully regulating its metabolic pathways (Escoll and Buchrieser, 2019). Therefore, enzyme abundance and activity are highly dependent on the growth environment. Although much progress has been made in understanding central metabolic pathways that are constitutively active, significant gaps in our knowledge still exist for condition-specific metabolic reactions (Cesur et al., 2022). The lack of knowledge about the function of condition-specific enzymes limits our understanding of cell biology as well as the cellular processes involved in development and disease (Yuan et al., 2024).

The baker's yeast, *Saccharomyces cerevisiae* (from here on termed yeast), is a powerful model organism for studying the function of conserved enzymes because it shares most of its proteome with humans (Cohen et al., 2022; Kachroo et al., 2022), and it is easy to grow and genetically manipulate (Vanderwaeren et al., 2022). Indeed, historically, yeast was instrumental in discovering enzymes in many of the central and conserved metabolic pathways, widening our understanding of cellular metabolism (Caudy et al., 2013; Kohlhaw, 2003; Alifano et al., 1996).

However, even in the well-studied yeast, hundreds of enzymes are still uncharacterized, comprising ∼20% of the enzyme repertoire (Cohen et al., 2022). Although there are many underlying causes, a central one is the narrow grid of growth conditions under which most of the research is performed. Indeed, whereas ‘central’ metabolism, such as the glycolysis pathway (Lao-Martil et al., 2022), has been well studied, ‘peripheral’ metabolism, such as that activated in unique conditions, remains less explored.

Harmful or extreme environmental conditions can damage vital cellular components leading to cellular stress and pathologies. Therefore, it is important to understand the mechanisms providing tolerance and adaptation to these stresses (Yuan et al., 2024). Identifying the enzymes that play a role in such conditions is central to better understanding biology under extreme environments. For example, take Gpd1, a key metabolic enzyme that catalyzes the conversion of dihydroxyacetone phosphate (DHAP) into glycerol-3-phosphate in the glycerol biosynthesis pathway. Although it is expressed even under standard growth conditions, during osmotic stress, yeast cells upregulate Gpd1 levels to produce more glycerol, balancing internal osmotic pressure (Albertyn et al., 1994; Pallapati et al., 2022). Hence, during conditions where Gpd1 activity becomes essential, its protein levels are upregulated. Therefore, by mapping conditions under which enzyme levels increase, we stand to gain insight on both their enzymatic and cellular activity (Lin et al., 2022).

While transcriptional changes for the entire yeast mRNA pool have been mapped under hundreds of conditions (SPELL database: Hibbs et al., 2007), a similar undertaking has not been performed at the protein level.

To follow upregulation of enzymes at the protein level, we created a pipeline for tracking protein abundance under diverse conditions. To this end, we assembled a collection of yeast strains, each representing one putative uncharacterized enzyme fused to a fluorophore and under the control of their native promoter. We subjected the strains to various stresses and extreme metabolic environments, uncovering conditions inducing the expression for 16 of them. Out of those we focused on a poorly characterized alcohol dehydrogenase, Bdh2, whose expression was induced under extreme nutrient limitations. We found that loss of Bdh2 had profound implications on the resistance of yeast to these conditions. We manipulated the expression levels of Bdh2 and obtained metabolic fingerprints of the cells under the applied condition. This allowed us to suggest several cellular functions of Bdh2. To biochemically validate these effects as primary activities of Bdh2, we purified the protein and reconstituted its activity *ex vivo*. These findings enhance our understanding of enzyme roles within the cell, alter our understanding of yeast metabolism during stress and have implications for biotechnology.

## RESULTS

### Screening a collection of putative enzymes under diverse conditions uncovers environments where they are upregulated

Uncovering the function of uncharacterized enzymes will deepen our understanding of metabolic networks. To study an enzymatic function, first, we must find a set of conditions where the protein is active. Although this is impossible for enzymes whose activity is unknown, we can use a proxy – uncovering conditions where protein levels are upregulated, suggesting the requirement of that protein and its activity. To approach this task systematically, we utilized a pre-existing collection (Valenti et al., 2025) and assembled yeast strains from it, each genomically encoding one uncharacterized enzyme, fused on its C-terminus to GFP. This genomic manipulation leaves the protein expressed from its natural endogenous promoter. Out of the 282 uncharacterized proteins predicted to have enzymatic function (Cohen et al., 2022), we picked 179 that were correctly represented in the collection (Valenti et al., 2025), in addition to characterized proteins as controls (Table S1).

**Fig. 1.**
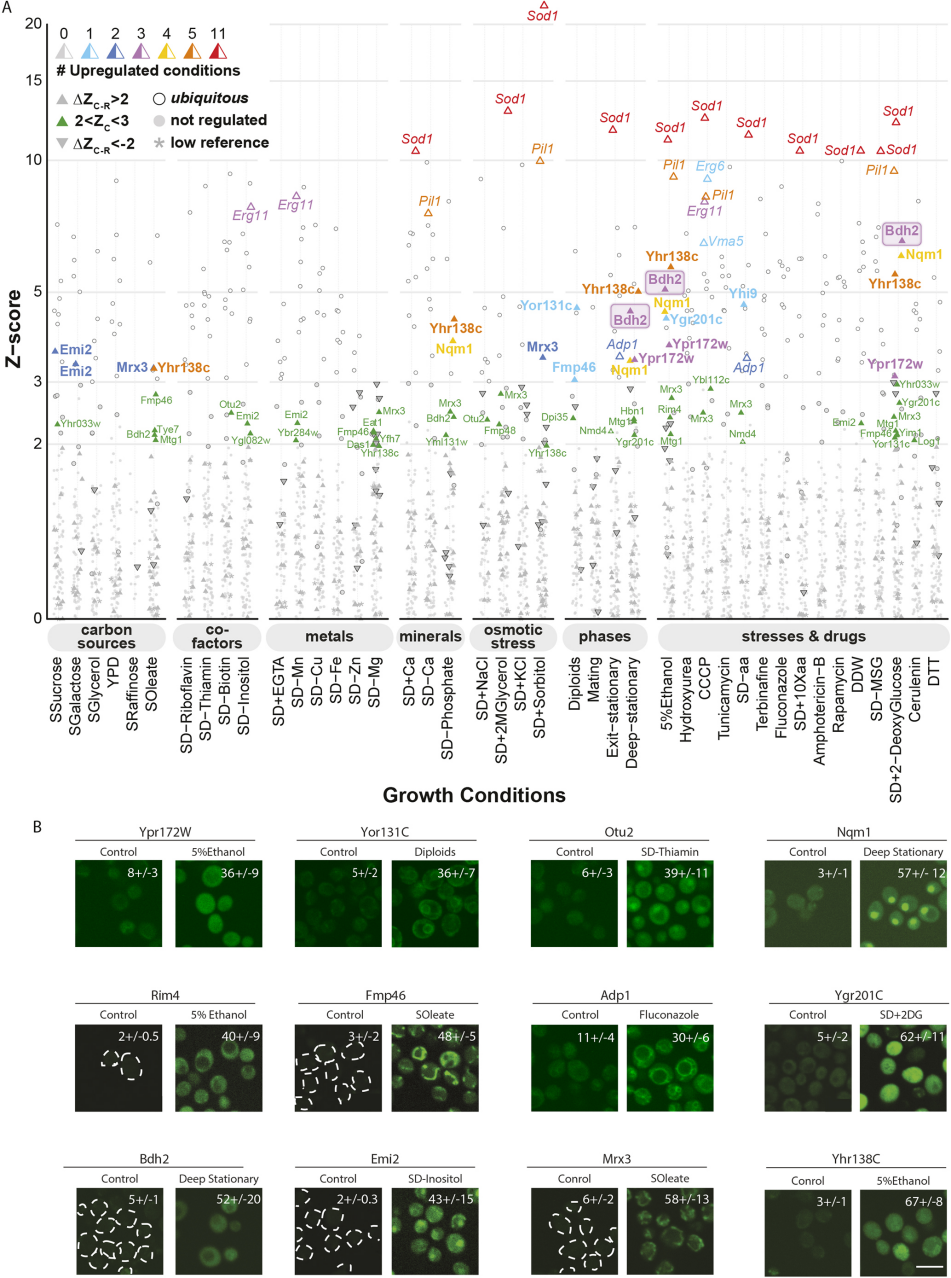
**Screening multiple environments uncovers condition-induced proteins.** (A) Dot plot of normalized protein expression per strain, reported as the fluorescence intensity *Z*-score in each condition, (*Z*_C_, *y*-axis) across growth conditions on the *x*-axis. Conditions are grouped by category: carbon sources, cofactors, metals, minerals, osmotic stresses, growth phases, stresses and drugs. Condition-dependent change relative to that for cells in reference medium is encoded by the difference in *Z*-scores between condition and reference medium (Δ*Z*=Z_C_−Z_R_). Upward triangles corresponds to increased condition-dependent expression (ΔZ>2); downward triangles indicate decreased expression relative to reference (Δ*Z*<−2), and circles denote strains exhibiting modest or no changes (|ΔZ|≤2). Strains whose signal in reference medium is below background are marked with a star. Strains with ubiquitously high expression across conditions are shown with transparent fill and a black outline to indicate non-significant induction. ‘Primary hits’ (see Materials and Methods) are colored by the number of conditions in which the tagged protein is upregulated (*Z*_C_≥3 and Δ*Z*>2, scale above) but also induced with respect to the reference condition. Labels are bold for non-ubiquitous primary hits and italic for ubiquitous primary hits. Potential hits (ΔZ>2 with 2<ZC<3) are shown as green upward triangles with the strain name annotated in green. The median fluorescence intensity for each strain was calculated from hundreds of cells from a single biological replicate (*n*=1); for background correction, results from eight biological replicates of non-fluorescent strains were aggregated. (B) Fluorescence microscopy images of strains in one example condition compared to the expression in reference control condition. Mean fluorescent intensity (mean±s.d.) across cells is reporter on the top right corner of the images (*n*=two biological repeats for each of the 12 strains). Scale bar: 5 μm.

To identify conditions where putative enzymes are upregulated, we grew the strains in 42 different conditions, including various carbon sources, nutrient changes, growth phases, drugs and stresses (Table 1; Table S2). We analyzed the fluorescence intensities of the strains in each condition. Our quantitative pipeline based on a dual-scoring approach (see Materials and Methods) yielded 46 primary hits of high-confidence, reflecting their strong condition-specific induction, even relative to the reference condition (Fig. 1A). Although half of the hits originated from enzymes with ‘ubiquitous’ high expression among most conditions, their significant induction relative to the reference condition suggests that they are specifically triggered by those stress conditions.

**Table 1.** Conditions screened for unique expression patterns

Stresses and drugs	Carbon sources	Metals	Co-factors	Osmotic stress	Phases	Minerals
SD +2DG	SGlycerol	SD −Fe^2+^	SD −thiamin	SD +KCl	Exit stationary	SD −Ca^2+^
5% ethanol	SGalactose	SD −Cu^2+^	SD −inositol	SD +NaCl	Deep stationary	SD +Ca^2+^
DDW	SSucrose	SD −Zn^2+^	SD −riboflavin	SD +sorbitol	Mating	SD −phosphate
SD −aa	SRaffinose	SD +EGTA	SD −biotin	SD +2 M glycerol	Diploids	
SD +10× aa	SOleate	SD −Mg^2+^				
DTT	YPD	SD −Mn^2+^				
SD no MSG						
Hydroxyurea						
Fluconazole						
Amphotericin B						
Terbinafine						
Tunicamycin						
CCCP						
Rapamycin						
Cerulenin						

A table showing the conditions used to screen the strains for protein expression, clustered by type of perturbation. In the carbon source column, the ‘S’ indicates the minimal growth medium for yeast immediately followed by the carbon source substituting for the dextrose present in standard SD medium. Abbreviations: SD, synthetic dextrose; DG, deoxyglucose; DDW, double distilled water; aa, amino acids; DTT, dithiothreitol; MSG, monosodium glutamate; CCCP, carbonyl canide m-chlorophenyl hydrazone; YPD, yeast extract peptone dextrose.

In total, we identified 16 upregulated proteins among 20 different conditions, including six ‘ubiquitous’ enzymes that served as positive controls. Some of those proteins were induced in multiple conditions, but some only in a single one (Fig. 1A). For example, the positive control Sod1 (a superoxide dismutase) was upregulated in 11 conditions relative to the reference, consistent with its role in detoxifying superoxide and the broad engagement of redox homeostasis across diverse stresses (Gasch et al., 2000). On the other hand, the induction of Yor131c (a putative haloacid-dehalogenase-like hydrolase) only stood out in the diploid phase in line with the report of increased abundance under DNA-replication stress, such as in ploidy-linked cell-cycle control (Niu et al., 2008).

Conversely, some conditions did not significantly affect the expression of any candidate, whereas others impacted the expression of many proteins, such as 5% ethanol (seven hits) and 2-deoxyglucose (six hits). Overall, the microscopy and analysis pipelines provided accurate results given that all the 12 strains that we picked for manual validation and verification demonstrated protein upregulation (Fig. 1B).

### Bdh2 is a putative alcohol dehydrogenase that has a central role under nutrient-limiting conditions

**Fig. 2.**
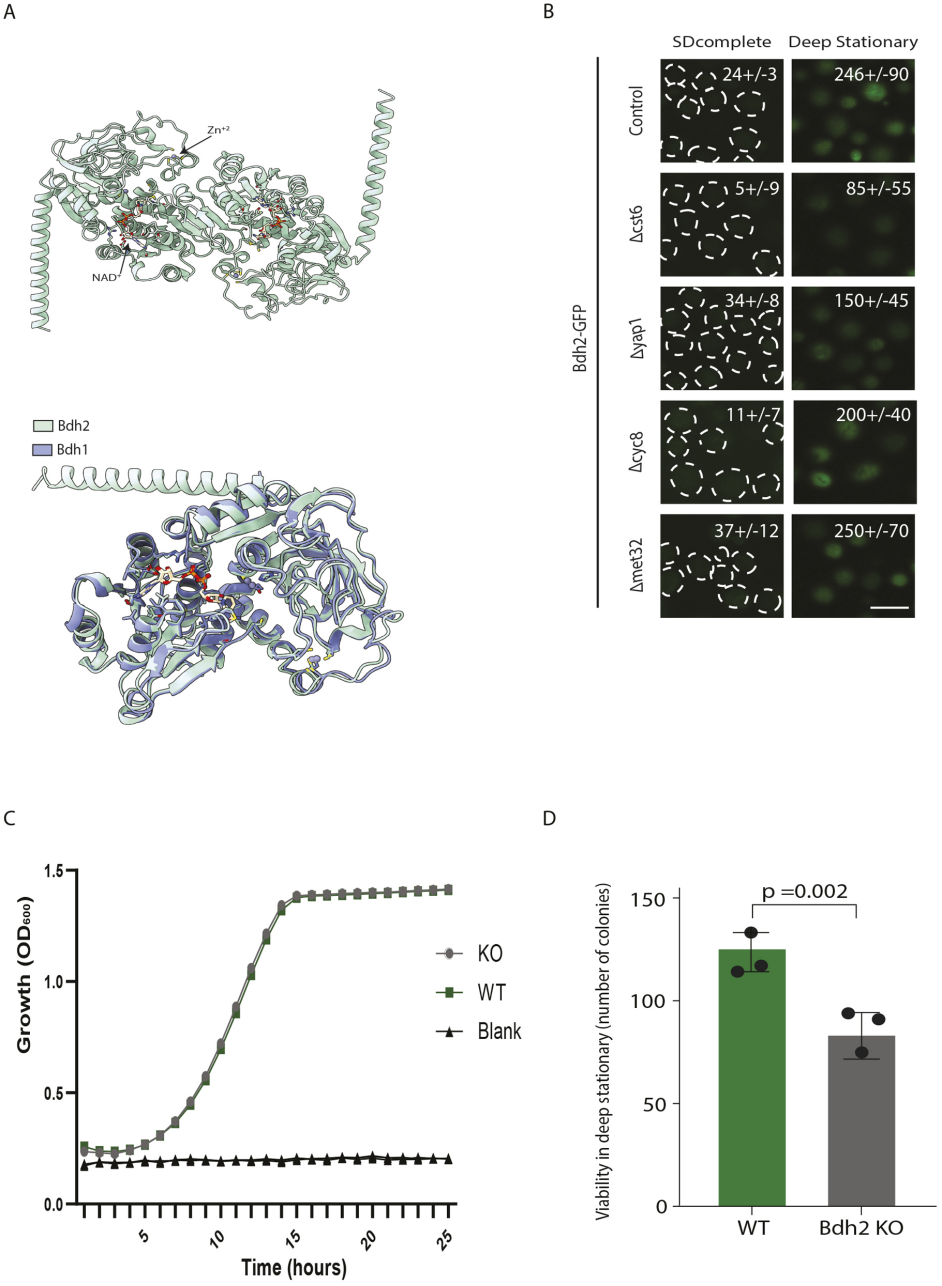
**Bdh2 is upregulated by Cst6 and has a unique role under extreme starvation conditions.** (A) An AlphaFold model of Bdh2 along with the Zn^2+^ and NAD^+^ co-factors, indicated by the arrows. The structure overlays the AlphaFold structures of Bdh2 and its characterized alcohol-dehydrogenase paralog Bdh1. Protein structural models were computed using AlphaFold3 (Abramson et al., 2024) using the reference protein sequence of Bdh2 from the Saccharomyces Genome Database (SGD) as the input. (B) Fluorescence images that show the effect of the loss of several suggested regulatory transcription factors on the expression of Bdh2 in deep stationary phase. Mean fluorescence intensity following quantification appears on the top right of the images (mean±s.d.; *n*=10). Scale bar: 5 μm. (C) A growth curve of a control strain compared to strains deleted for Bdh2, in triplicates. Error bars are present but not clearly visualized due to their small size. (D) A bar graph showing the viability rates for the deletion of Bdh2 after growth to deep stationary phase relative to control, mean of 83 and 125, respectively (*n*=3). *P*-value of 0.002 in two sample *t*-test. WT, wild type; KO, knockout.

Out of the putative enzymes that were upregulated, we focused on an interesting candidate, Bdh2. In our screen, it qualified as a non-ubiquitous primary hit in three conditions (5% ethanol, deep stationary phase and 2-deoxyglucose). Bdh2 and its close paralog, Bdh1 (Fig. 2A), show sequence similarity to alcohol dehydrogenases and possess a putative alcohol dehydrogenase domain (Nash et al., 2020). Accordingly, the UniProt database (Coudert et al., 2023) suggests an Enzyme Commission (EC) number of 1.1.1.303 for Bdh2, which is butanediol (an alcohol) dehydrogenase activity. In support of this, it has been demonstrated that even in the absence of Bdh1, Bdh2 affects the degradation of the non-endogenous substrate vanillin, a phenolic aldehyde susceptible to reduction to alcohol by an alcohol dehydrogenase (Ishida et al., 2016). Although the cellular function of Bdh1 has been characterized (González et al., 2010), the endogenous substrates or products of Bdh2 have not been clearly demonstrated, nor has its physiological role. Amongst the many cellular alcohol dehydrogenases that have been extensively studied, the role of Bdh2 has remained unexplored.

Our data demonstrate that Bdh2 is upregulated during deep stationary phase, a condition where nutrients become highly limited. How then is Bdh2 regulated under these conditions? Several systematic studies have predicted transcription factors for Bdh2 (Hu et al., 2007; Carrillo et al., 2012; Venters et al., 2011). To test whether its upregulation is dependent on their activity, we followed the upregulation of Bdh2 on the background of their deletions. We found that loss of the transcription factor Cst6 resulted in the greatest reduction on Bdh2 expression (Fig. 2B). Interestingly, Cst6 is a Basic leucine zipper (bZIP) transcription factor from the ATF/CREB family involved in stress-responsive regulatory networks and has been shown to be involved in utilization of non-optimal carbon sources (Garcia-Gimeno and Struhl, 2000), such as those that would need to be exploited in deep stationary phase.

Given that Bdh2 is highly upregulated in this unique condition, we assayed whether it has a physiological role specific to deep stationary phase. Indeed, whereas loss of Bdh2 had no effect on yeast growth during logarithmic phase where nutrients are abundant (Fig. 2C), loss of Bdh2 resulted in a significant reduction of viability (manifested by the number of viable colonies) after exposure to deep stationary phase (Fig. 2D). The fact that Bdh2 is transcriptionally regulated by Cst6 and its clear role in maintaining viability during the nutrient limiting conditions of deep stationary growth, highlights Bdh2 as a key player in metabolic adaptation to extreme conditions.

### Analysis of metabolic changes induced by altering Bdh2 expression provides clues to its enzymatic activity *in vivo*

One way to study the activity of condition-specific uncharacterized enzymes would be to deplete them specifically under the extreme conditions in which they are usually upregulated and assay the effects of this manipulation. In anticipation of this, we had already assembled the strains assayed above from a collection that had, in addition to GFP, a tag enabling rapid protein degradation (Valenti et al., 2025). This tag encodes for the improved auxin-inducible degron system (AID2) (Yesbolatova et al., 2020). These strains are therefore unique in allowing us both to track protein abundance and localization as well as rapidly depleting them by adding a small auxin-like molecule (5-Ph-IAA). We have previously shown that the activities of the E3 ligase OsTIR1 (a plant protein from the rice *Oryza sativa*) (F74G), and the ubiquitin-proteasome pathway (that leads to protein depletion after 5-Ph-IAA addition) are widespread and therefore do not usually constrict the use of this method under various conditions (Valenti et al., 2025). To verify that this also holds true for the specific proteins and conditions that we uncovered (Fig. 1A), we tracked the most induced fusion proteins and assayed their degradation under the condition in which they were most upregulated. We found degradation for five of them (Fig. S1). Given that a control protein was degraded in all conditions (data not shown), this suggests that the proteins that were not degraded were specifically protected from degradation either by being in a unique cellular niche or by their fold or complex formation. However, Bdh2 could be rapidly degraded under deep stationary phase, allowing us to assay it using this method (Fig. S1A,B).

**Fig. 3.**
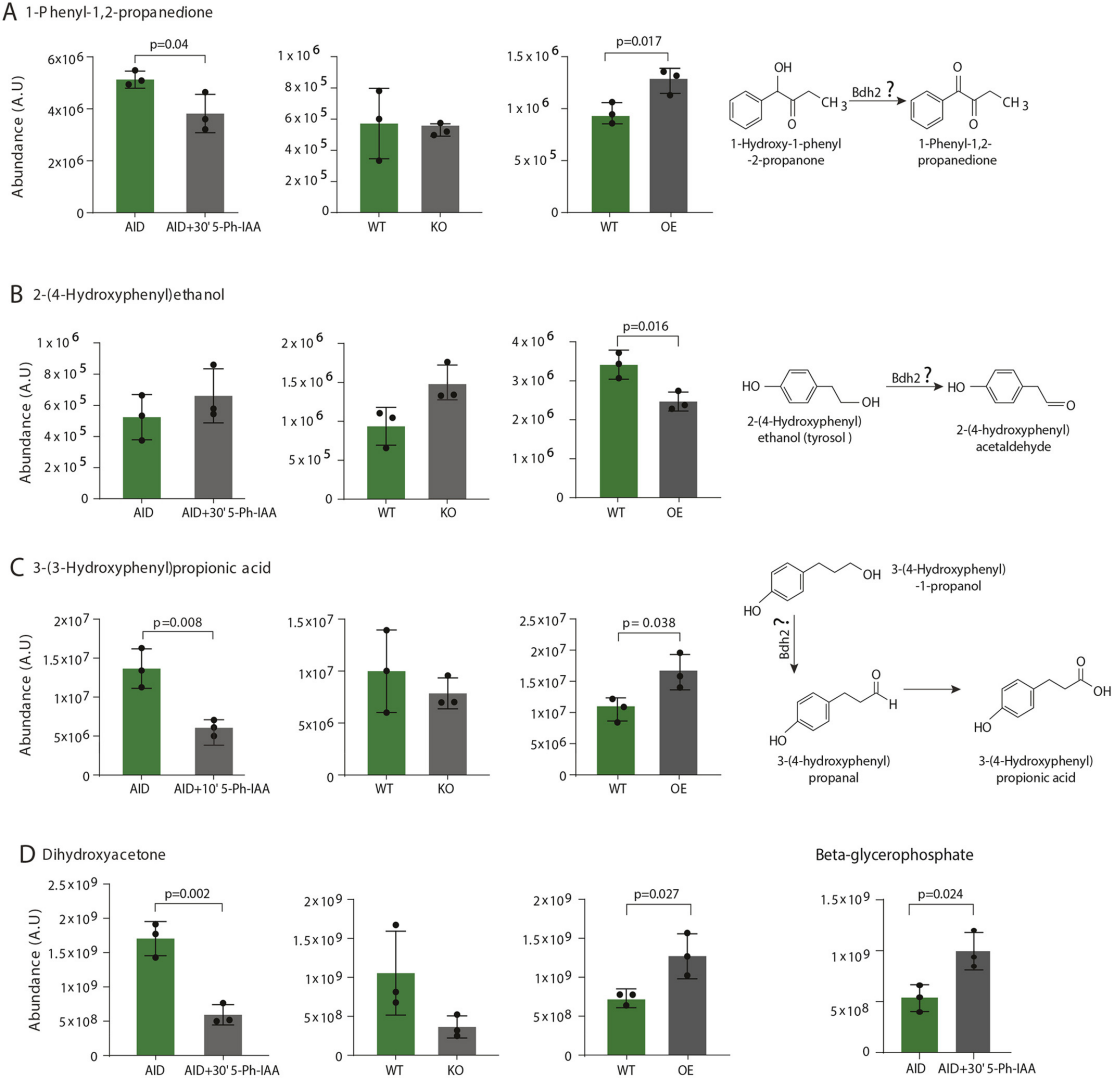
**Metabolomics analysis suggests a role for Bdh2, a predicted alcohol dehydrogenase.** (A) Bar graphs showing the changes in the ketone 1-phenyl-1,2-propanedione upon depletion (AID), knockout (KO) and overexpression (OE) of Bdh2 compared to control. Right, the proposed reaction in which 1-phenyl-1,2-propanedione takes part. (B) Bar graphs showing the changes in the aromatic alcohol 2-(4-hydroxyphenyl)ethanol upon depletion, knockout and overexpression of Bdh2 compared to control. Right, the proposed reaction in which 2-(4-hydroxyphenyl)ethanol takes part. (C) Bar graphs showing the changes in the acid 3-(4-hydroxyphenyl)propionic acid upon depletion, knockout and overexpression of Bdh2 compared to control. Right, the proposed reaction in which 3-(4-hydroxyphenyl)propionic acid takes part. (D) Bar graphs showing the changes in the ketone dihydroxyacetone upon depletion, knock out, and overexpression of Bdh2 compared to control. *P*-values from *t*-test of two-tailed distribution appear in all graphs with significant changes. All results are mean±s.d.; *n*=three biological repeats for each of the 26 strains. WT, wild type; A.U., arbitrary units.

We therefore continued to assay the metabolic fingerprint of altering Bdh2 expression by depleting Bdh2 using the AID2 system under the condition in which Bdh2 abundance increased. In parallel, we either deleted or overexpressed Bdh2 constitutively. We utilized metabolic profiling (metabolomics) to measure the changes in putative metabolites between the various states (Tables S3, S4). As Bdh2 has a predicted alcohol dehydrogenase domain, we focused on changes related to alcohols, aldehydes (precursors to alcohols), and ketones (the products of alcohol dehydrogenation). Indeed, we found four significantly altered putative substrates or products from these groups. First phenyl-1,2-propanedione, a ketone, was significantly reduced when Bdh2 was depleted and elevated when it was overexpressed (Fig. 3A). Second, the aromatic alcohol 2-(4-hydroxyphenyl)ethanol, whose levels were decreased when Bdh2 was over-expressed. In addition, we noticed that during Bdh2 depletion (either in the deletion or in the auxin-mediated depletion), its levels increased, although this was not statistically significant (Fig. 3B). Third, the levels of the aldehyde 3-(4-hydroxyphenyl)propionic acid were significantly increased in the overexpression strain and reduced in the depleted strains (Fig. 3C). Finally, the most striking metabolite that was altered following changes in Bdh2 expression was the ketone dihydroxyacetone. Its levels were markedly reduced when Bdh2 was depleted and elevated when Bdh2 was overexpressed. In addition, β-glycerophosphate (also known as glycerol-2-phospate or G2P) was significantly increased following 5-Ph-IAA-induced degradation of Bdh2, which was interesting considering that glycerol (an alcohol) dehydrogenation would produce hydroxyacetone (a ketone) (Fig. 3D). Interestingly, some compounds did not change significantly in the deletion strain but did in the depletion conditions. This most likely stems from the extensive metabolic rewiring to maintain homeostasis that cells undertake following a genetic alteration. This underlines the importance of using an ‘on-demand’ degradation system. Regardless, the metabolic changes suggest potential reactions that could be carried out by Bdh2 *in vivo* during survival in nutrient-limiting conditions. However, as metabolism is so intricately interconnected, *in vivo,* it is extremely difficult to differentiate direct from indirect effects of enzyme loss.

### Metabolic analysis of purified Bdh2 gives a clearer indication of its function

**Fig. 4.**
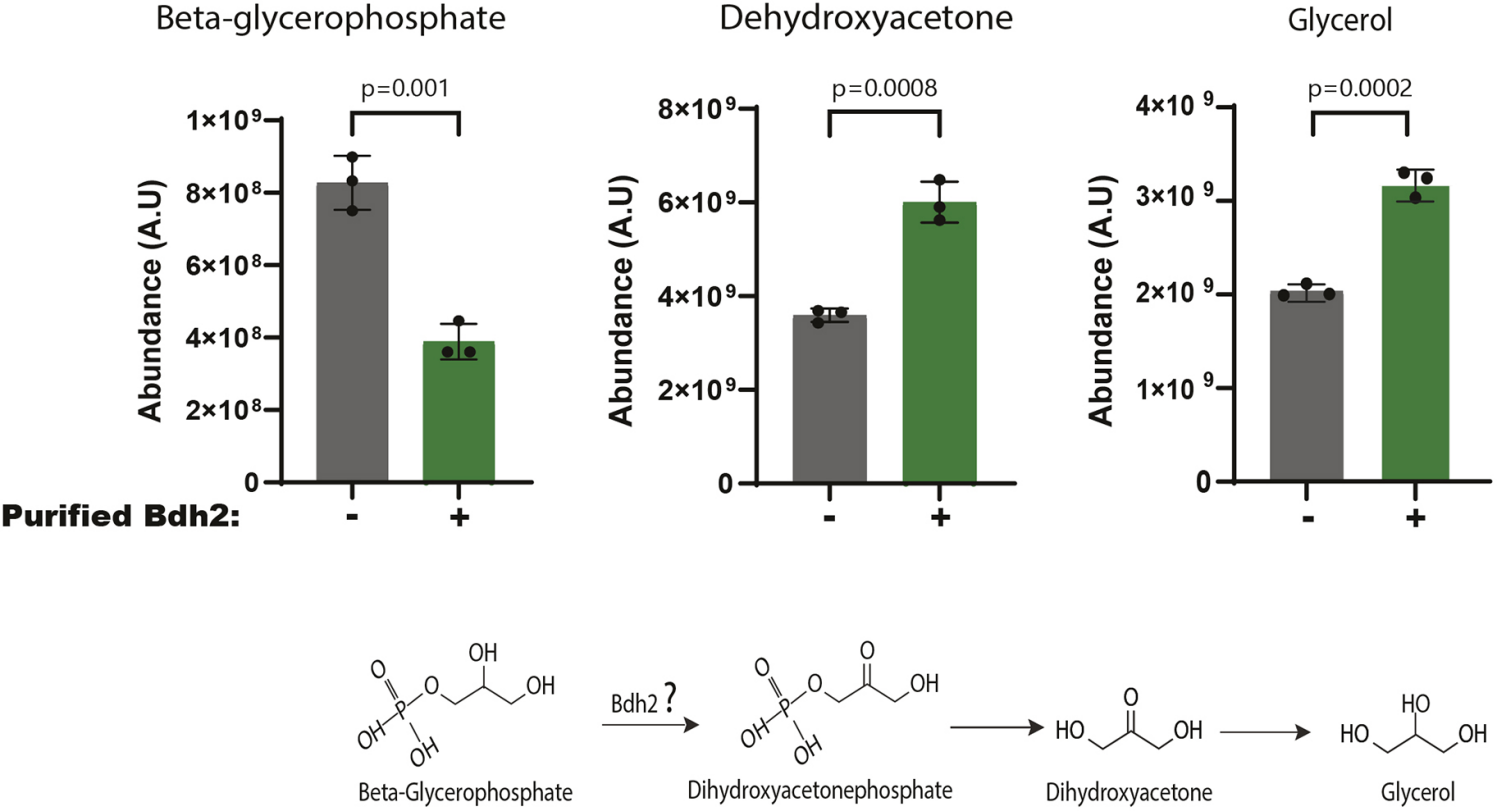
**Metabolomic analysis of purified Bdh2 suggests a direct role for Bdh2 in glycerol metabolism.** Bar graphs show the significant changes in metabolites upon addition of purified Bdh2 to cell extracts compared to buffer without Bdh2. Below, the metabolic pathway in with Bdh2 activity is suggested. *P*-values from *t*-test with two-tailed distribution are shown. All results are mean±s.d.; *n*=three repeats for each condition. A.U., arbitrary units.

To gain deeper insight into the direct activity of Bdh2, we purified the protein and prepared metabolic cell extracts from a Bdh2-depleted yeast strain. We then added the purified Bdh2 protein to the extract and compared the resulting metabolite profile to a control extract without the added protein (Table S5). Focusing again only on alcohols, ketones and aldehydes, our metabolomic analysis revealed a significant decrease in beta-glycerophosphate levels upon Bdh2 addition, accompanied by a notable increase in dihydroxyacetone and glycerol. These findings suggest that Bdh2 is directly and functionally involved in the glycerol metabolic pathway (Fig. 4). During carbon limitation, when cells must utilize non-conventional carbon sources, Bdh2 might play a beneficial role by enabling the cell to recycle β-glycerophosphate into metabolically useful intermediates.

## DISCUSSION

This study aimed to create a conceptual pipeline for discovering functions of uncharacterized enzymes in yeast that become active and important under specific environmental or stress conditions. Our pipeline provides a systematic route to identify condition-induced enzymes by quantifying protein-level responses across a broad environmental panel. Key design choices increased robustness: background subtraction using non-GFP controls, robust Z-scoring based on per-condition medians and median absolute deviation (MAD), and explicit exclusion of ubiquitous high-expressors and unquantifiable expression from the normalization reference. Together, these steps prevented false or misleading signals from biasing the *Z*-score normalization, thus preserving sensitivity for detecting genuine condition-specific upregulation.

Using a mini collection of tens of strains, we identified putative enzymes that are upregulated across many distinct conditions, such as nutrient deficiencies, oxidative stress and exposure to metabolic inhibitors. The screen yielded 46 high-confidence hits, mapping to 16 proteins that were induced in one or more conditions, with both generalist and specialist behaviors. We also noticed 44 potential hits that are induced relative to reference medium (ΔZ>2) yet not above the condition-specific threshold (2<*Z*_C_<3). None of those strains were ubiquitously highly expressed among conditions. Hence, these proteins are likely ‘off’ or expressed at low levels that become detectably induced under stress. Several of these enzymes also appeared as primary hits in other conditions (for example, Bdh2, Mrx3, Emi2 and Yhr138c), consistent with condition-dependent penetrance near our stringent *Z*-score threshold. We focused our main analysis on the dual threshold set to prioritize hits that combined strong induction with high absolute expression, while recognizing that these potential hits might contain biologically meaningful responses that merit targeted follow-up (Table S6).

We focused on one putative enzyme, Bdh2, whose expression was induced during growth in nutrient-deprived conditions. We found that loss of Bdh2 in these conditions dramatically reduces yeast viability. Using metabolomic analysis on strains in which we altered Bdh2 expression or with purified enzyme, we suggest the first biological roles for Bdh2.

Despite these significant findings, many enzymes in the library were not upregulated under any environment, and some were never visualized, even though we assayed 42 different conditions. Although several factors may contribute to this, we hypothesize that some of the enzymes are expressed at very low levels, which makes them difficult to detect using fluorescence microscopy due to the inherent autofluorescence of the yeast cells. Some conditions might have induced an expression that is functionally significant but cannot be detected by fluorescence microscopy. To address these challenges, future studies could involve alternative tagging strategies or explore broader environmental conditions.

We focused on Bdh2 and found that it could have a unique role in conditions where nutrients are sparse. Its regulation by Cst6 suggests that it might specifically be important for dealing with alternative carbon sources. Indeed, one putative metabolite that showed coherent changes was 2-(4-hydroxyphenyl)ethanol, also known as tyrosol, a fusel alcohol formed as part of the Ehrlich pathway (Ehrlich., 1907). The catabolism of amino acids is a valuable source of nitrogen during starvation and is a multi-enzyme process (Hazelwood et al., 2008). However, the specific contributions of each enzyme under various physiological conditions in the Ehrlich pathway are not fully delineated (Hazelwood et al., 2008). Unfortunately, this compound was not detected at all in our *ex vivo* metabolic analysis with purified Bdh2. Hence, we cannot say with certainty if it is indeed a direct substrate, but this remains an enticing hypothesis. Further research is needed to verify whether Bdh2 could indeed be one of the alcohol dehydrogenases acting on the Ehrlich pathway.

The *ex vivo* analysis with purified enzyme supports a direct role for Bdh2 in the glycerol pathway. First, we consistently see that alterations in Bdh2 impacted dihydroxyacetone. Loss of Bdh2 led to its reduction whereas overexpression or addition of purified protein led to its accumulation – suggestive of this metabolite being a product of Bdh2 activity. Dihydroxyacetone can be converted by an alcohol dehydrogenase directly from glycerol, a byproduct of phospholipid catabolism (Klein et al., 2017). However, our data do not support direct conversion, as glycerol levels increased upon the addition of purified Bdh2. Rather, we saw a consistent effect on β-glycerophosphate – its levels decreased after the addition of Bdh2 and were doubled after only 30 min of Bdh2 degradation using the AID2 system. In conditions of nutrient scarcity and urea accumulation (Zhu et al., 2020), β-glycerophosphate (likely glycerol-2-phosphate) can appear as a side-product, but it cannot substitute for glycerol-3-phosphate as a backbone for triglyceride synthesis and is therefore considered a metabolic dead end. To re-enter central metabolism, β-glycerophosphate would need to be hydrolyzed to glycerol and then re-phosphorylated to glycerol-3-phosphate via the canonical pathway. However, its regulated formation and physiological role remain unclear.

Our working hypothesis is that Bdh2 has a unique role in directly degrading β-glycerophosphate to DHAP. This is quite unique as alcohol dehydrogenases rarely work on phosphorylated substrates directly and this might suggest why this enzyme evolved. Once the phosphorylated dihydroxyacetone is formed, it can be dephosphorylated by dedicated enzymes, and glycerol will then be produced by the inverse activity, through hydrogenation. Although our metabolite prep included a denaturation step for proteins, some proteins might have refolded during the 1-h incubation time with purified Bdh2, explaining the Bdh2-independent activities we observed. If this is the case, Bdh2 plays a beneficial role by enabling the cell to recycle β-glycerophosphate into metabolically useful intermediates.

Regardless of the specific substrate or product, yeast mostly respire during nutrient-limiting conditions (Tu et al., 2007). Thus, the expression of Bdh2 might have specific importance under these severe starvation conditions. In contrast, Bdh1, the paralog of Bdh2, is constitutively expressed, potentially serving a housekeeping role in alcohol metabolism under normal growth conditions, continuously contributing to fermentation and energy turnover (Nash et al., 2020).

Overall, our study identified putative enzymes that are induced under specific environmental conditions. Yet, it is important to acknowledge potential methodological limitations in the data analysis and experimental design. Future methodological improvements could further enhance the power and robustness of the screen. Specifically, the strategy of using fixed thresholds for hit detection implicitly assumes a similar level of biological and technical variability across all conditions. While the robust *Z*-scoring helps to mitigate this, condition-specific differences in noise might affect the sensitivity for detecting hits in certain environments. For example, increasing the number of biological replicates per strain and condition could further reduce biological noise. Furthermore, implementing an adaptive thresholding strategy for the Δ*Z*-score (Δ*Z*) could be used to dynamically adjust the hit-detection threshold based on the measured level of noise in each specific condition, thereby improving the overall sensitivity and rigor of the pipeline. Future work could also expand the condition panel or incorporate orthogonal quantification (for example, targeted proteomics). However, we bring here 12 manually validated enzymes with confirmed upregulation, and our case-study analysis of Bdh2 illustrates how such a screen can guide mechanistic follow-up under nutrient limitation.

Our work therefore provides a roadmap for future research into the characterization of condition-specific enzymes, which might play a specific role in yeast survival under stress. This promotes our efforts to uncover more enzyme functions and should advance our understanding of cellular metabolic networks and their regulation.

## MATERIALS AND METHODS

### Yeast strains

Overall, we assembled a collection of 184 *S. cerevisiae* strains, each bearing a C-terminal GFP fusion at the endogenous locus of a putative enzyme and expressed from its native promoter. In addition, eight non-fluorescent control strains were included, yielding 192 strains in total. All the strains are listed in Tables S1 and S7. All primers were designed using the Primers-4-Yeast web tool (Yofe and Schuldiner, 2014) (Table S8).

### Yeast growth

Yeast cells were grown on solid medium containing 2.2% (w/v) agar (Formedium #AGA0X) or liquid medium, at 30°C. Antibiotics used were: nourseothricin (NAT, WERNER BioAgents ‘ClonNat’ #5.XXX.000) at 0.2 g/l and geneticin (G418, Formedium #G418X) at 0.5 g/l for strain maintenance and overnight pre-cultures. Unless stated otherwise, the medium used was synthetic dextrose minimal medium [SD; 0.67% (w/v) yeast nitrogen base (YNB) without amino acids or ammonium sulfate (Formedium #CYN04XX), 0.1% L-glutamic acid monosodium salt, 2% (w/v) glucose], supplemented with amino acid OMM mix (Hanscho et al., 2012) [SD (MSG) Complete]. For the expression tests and microscopy, the strains were grown in conditions with different media (Table 1; Table S2).

### Quantification and analysis of fluorescence microscopy data

We analyzed fluorescence microscopy data to identify uncharacterized enzymes whose relative expression increased under diverse environmental conditions. Yeast strains expressing fluorescently tagged enzymes were grown separately in 42 stress conditions plus a reference condition (SD medium). Each plate contained 184 fluorescent strains and 8 non-fluorescent controls for background estimation. The dataset comprised 8243 strain–condition measurements (192 positions×43 conditions), accounting for 13 growth dropouts. Following image acquisition, the microscope software segmented cell boundaries and returned the median and standard deviation of single-cell fluorescence intensity. We processed the data through the following pipeline:

1. Strains with low cell counts (<30 detected cells) or high cell-to-cell variability (coefficient of variation>100%) were excluded.
2. Background fluorescence was estimated per plate and condition from the median intensity of the 8 non-fluorescent controls and subtracted from the measured intensities of the fluorescent strains.
3. Background-corrected intensities were floored to 1.0 to avoid negative values and log_2_-transformed.
4. To avoid distortion by constitutively bright strains, we identify 11 ‘ubiquitous’ enzymes with median percentile across conditions above 0.9 (*n*=460 strain–condition measurements). We also flagged strains with non-quantifiable signal (*n*=377 strain-condition intensity below background). Both groups were excluded from expression normalization.
5. Corrected intensities were normalized within each condition using a robust *Z*-score based on the median and median absolute deviation (computed on the remaining 6751 strains). providing relative expression per plate, thus mitigating between-plate effects.
6. A Δ*Z*-score quantified induction relative to the reference by calculating the difference between the condition-specific Z-score (*Z*_C_) and the *Z*-score in the reference condition (*Z*_R_). Δ*Z*=*Z*_C_ – *Z*_R_.
7. High-confidence hits met dual thresholds *Z*≥3 and Δ*Z*≥2.0. We distinguished primary hits (non-ubiquitous) from hits arising among ubiquitous high-expression strains.
8. We additionally reported potential hits (2<*Z*<3 and Δ*Z*>2) that did not originate from ubiquitous strains.

This approach identified uncharacterized enzymes with significant, condition-specific induction relative to both the plate population and the reference condition.

### Automated high-throughput fluorescence microscopy

For high-throughput fluorescence microscopy imaging, the strains were transferred from agar plates into 384-well plates for growth in 100 μl condition-specific medium (Table S2) overnight (ON) at 30°C using a RoToR arrayer (Singer). The ON culture was back diluted into 384-well plates in condition-specific medium to an optical density at 600 nm (OD_600_) of ∼0.2. After 4 h, 50 μl from each well was transferred to a glass-bottom 384-well microscopy plate (Azenta Life Sciences) coated with concanavalin A (Sigma-Aldrich). The cultures were allowed to adhere to the bottom of the plate for 20 min. After incubation, wells were washed with condition-specific medium to remove non-adherent cells, all was done in EVO freedom liquid handler (TECAN). The plates were then transferred to an Olympus automated inverted fluorescent confocal microscope system using a robotic swap arm (Peak Analysis & Automation). Cells were imaged at room temperature (RT) in condition-specific medium using a 60× air lens (NA 0.9) and with an ORCA-flash 4.0 digital camera (Hamamatsu). Images were recorded with 488 nm laser illumination for the GFP channel (excitation 488 nm, emission filter B525/50 nm), with 600 ms exposure and 60% laser intensity, mild conditions to minimize photobleaching. Bright-field images were also taken, with 200 ms exposure and 100% halogen bulb. Four positions were imaged per well and the software autofocus was used to ensure the cells were imaged in their central plane for proper comparison (scanR version 3.2).

Images displayed in Figs 2 and 3 were acquired at RT using a VisiScope Confocal Cell Explorer system, composed of a Zeiss Yokogawa spinning disk scanning unit (CSU-W1) coupled to an inverted Olympus IX83 microscope with a 60× oil objective (NA 1.4). The excitation wavelength was 488 nm, with an exposure time of 600 ms and 80% LED intensity. Images were taken by a PCO-Edge sCMOS camera controlled by VisiView 3.2.0 software.

The microscopy images were cropped and attributed with a color gradient for representing the range of fluorescence intensity in ImageJ (Schneider et al., 2012). The brightness and contrast of images were linearly adjusted so that all GFP images of the same strain (before and after induction) have the same parameters.

### 5-Ph-IAA-induced degradation assays

Protein depletion was assayed under some of the tested conditions (Fig. S1A) after 30 min of 5 μM 5-Ph-IAA (an auxin analog, Bio Academia: Product code: 30-003-10). Addition of 5 μM 5-Ph-IAA was undertaken in the lag phase (30 min before the back dilution time ends), and the cells were washed with medium plus 5 μM 5-Ph-IAA for imaging.

### Western blot analysis

Cells were grown ON in rich medium [YPD; YPD broth 5% (w/v) Formedium #CCM02XX: 2% (w/v) peptone, 1% (w/v) yeast extract and 2% (w/v) glucose] under antibiotic selection (NAT and G418) and used to inoculate 20 ml of YPD to OD_600_=0.05. Cells were incubated with shaking at 30°C until OD_600_=0.2, where they were split into two identical cultures. One culture received 5 μM 5-Ph-IAA (30 min before the final time point), and the other culture was treated with DMSO as control. Cells were harvested after 3.5 h, except for strains in double deionized water (DDW), which were incubated for another 4 h, and cells grown in deep stationary medium (i.e. a nutrient-depleted medium obtained after filtering out cells cultured for 48 h to deep stationary phase, when most nutrients have been consumed), which were incubated for 24 h, to detect the effect of the stress on protein expression. Cells were harvested by centrifugation (3000 ***g*** for 3 min) followed by flash freezing in liquid nitrogen. Protein extraction, SDS-PAGE, and western blotting were performed as described previously (Eisenberg-Bord et al., 2021). Briefly, the cells were lysed with 8 M urea-based lysis buffer with protease inhibitors by glass bead-beating. Lysates were denatured by the addition of SDS (final concentration ∼2%) and a 45°C incubation for 15 min. Denatured lysates were centrifuged to separate cell debris. Loading buffer containing DTT (final concentration ∼25 mM) was added, and samples were incubated at 45°C for 15 min. 30 μg sample was loaded onto 10% agarose gels and separated with electrophoresis, then transferred onto nitrocellulose membrane using the Trans-Blot Turbo transfer system (Bio-Rad). Membranes were blocked in SEA BLOCK buffer (Thermo Scientific), incubated with primary antibodies (anti-GFP, Abcam, ab290 1:1000, and anti-actin, Abcam, ab170325 1:5000), washed, and incubated with fluorescent secondary antibodies for 1 h (Li-COR, 926-32210, 1:10,000 and Abcam, ab216777, 1:10,000). After washing, the membranes were imaged on the LI-COR Odyssey Infrared Scanner (Fig. S1B).

### Viability assays

For the viability assays, the strains were grown ON in standard medium. Next, 10 ml cultures at an OD_600_ of 0.1 were incubated in triplicates or duplicates for 4 h in deep stationary medium (i.e. a nutrient-depleted medium obtained after filtering out cells cultured for 48 hours to deep stationary phase, when most nutrients have been consumed) and left in the incubator at 30°C. Then 10 μl of each culture, at time 0 and after 3 days, was diluted in 1 ml of DDW, and 100 μl dilution was plated on agar plates with selections. The plates were grown in 30°C for two days. After 2 days, the number of colonies from each plate was counted and compared between the depletion of Bdh2 and the control.

### Sample preparation for metabolomics

Starter cultures were prepared by inoculating 3 ml of SD complete medium, under antibiotic selection (NAT and G418), with each strain in triplicates and incubating ON at 30°C on a shaker. Then, 50 μl of the overnight cultures were used to seed 5 ml fresh cultures of SD complete or condition-specific media (without antibiotics) and incubated ON at 30°C on a shaker. Next, 50 ml cultures at an OD_600_ of 0.5 were incubated for 4 h and 5-Ph-IAA (5 μM final concentration) was added to half of the samples followed by a 30 min or 10 min incubation at 30°C. Samples were harvested by centrifugation (3000 ***g*** for 3 min), washed in 1 ml DDW, centrifuged, and after removal of the DDW, the pellets were snap-frozen in liquid nitrogen.

### Metabolite extraction

Extraction and analysis of polar metabolites were performed as previously described (Malitsky et al., 2016; Zheng et al., 2015), with some modifications: yeast cell pellets were extracted with 1 ml of a pre-cooled (−20°C) homogenous methanol–methyl-tert-butyl-ether (MTBE) 1:3 (v/v) mixture. The tubes were vortexed and then sonicated for 30 min in an ice-cold sonication bath (taken for a brief vortex every 10 min). Then, DDW–methanol (3:1, v/v) solution (0.5 ml) containing internal standard, C13, and N15 labeled amino acids standard mix (Sigma, 767964), was added to the tubes, followed by centrifugation (15,000 ***g*** for 10 min). The upper organic phase was transferred into a 2 ml Eppendorf tube. The polar phase was re-extracted as described above, with 0.5 ml of MTBE, and the organic phase was combined with the first extraction. The polar phase was moved to a new Eppendorf, dried for 1.0 h by N2 to remove organic solvents, and then lyophilized. Before the injection into the LC-MS machine, the polar phase sample pellets were dissolved using 150 μl DDW-methanol (1:1), centrifuged twice (15,000 ***g***) to remove possible precipitants, and transferred to an HPLC vial.

### LC-MS polar metabolite analysis

Metabolic profiling of the polar phase was performed as described (Zheng et al., 2015) with minor modifications described below. Briefly, analysis was performed using Acquity I class UPLC System combined with mass spectrometer Q Exactive Plus Orbitrap™ (Thermo Fisher Scientific), which was operated in a negative ionization mode. The LC separation was done using the SeQuant Zic-pHilic (150 mm×2.1 mm) with the SeQuant guard column (20 mm×2.1 mm) (Merck). The Mobile phase B was acetonitrile, and Mobile phase A was 20 mM ammonium carbonate with 0.1% ammonia hydroxide in DDW and acetonitrile (80:20, v/v). The flow rate was kept at 200 μl/min, and the gradient was as follows: 0–2 min 75% of B, 14 min 25% of B, 18 min 25% of B, 19 min 75% of B, for 4 min. Injection volume was 2 μl.

Mass spectral data were acquired within an *m*/*z* range of 70–1050 using heated electrospray ionization (HESI) in negative mode. Ion source parameters included capillary temperature at 325°C, spray voltage at 3.25 kV, sheath gas flow rate at 40, auxiliary gas flow rate at 10 (arbitrary units), and auxiliary gas temperature at 50°C. MS1 spectra were acquired at a resolution of 35,000 full width at half maximum (FWHM), with data-dependent MS/MS acquisition conducted using an isolation window of 3 *m*/*z* and a resolution of 17,500 FWHM.

### Statistical analysis of the metabolic data

For the statistical comparison between metabolite levels before and after protein depletion, a *t*-test with a two-tailed distribution and a two-sample equal variance (homoscedastic) were used. Presented tabs show basic statistical analysis (*t*-test) in Tables S3–S5.

### Cloning, expression, and purification of Bdh2

Bdh2 (*S. cerevisiae*) gene was amplified from yeast genomic DNA and cloned into the pET28a expression vector (Novagen) using Restriction-Free (RF) cloning (Unger et al., 2010), yielding an expression construct encoding an Bdh2 fused to C-terminal Strep tag. The construct was transformed into *E. coli* BL21(DE3) and cultured in lysogeny broth (LB) supplemented with 30 μg/ml kanamycin. A 5 l culture was grown at 37°C until reaching an OD_600_ of 0.6–0.8, followed by induction with 200 μM isopropyl β-D-1-thiogalactopyranoside (IPTG). Protein expression was carried out for 16–18 h at 15°C. Cell pellets (5400 ***g*** for 15 min at 4°C) were resuspended in lysis buffer containing 100 mM Tris-HCl pH 8.0, 150 mM NaCl, and 1 mM EDTA, supplemented with 0.2 mg ml^−1^ lysozyme, 20 μg ml^−1^ DNase I, 1 mM MgCl_2_ and a protease inhibitor cocktail (Calbiochem Set III). Cells were lysed using a cooled Constant Systems cell disruptor, and the soluble fraction was clarified by centrifugation (20,000 ***g***, 20 min, 4°C) followed by filtration through a 0.22 μm membrane. The clarified lysate was applied to a pre-equilibrated 5 ml Strep-Tactin® XT column (IBA Lifesciences) at a flow rate of 1 ml min^−1^ using an ÄKTA FPLC system (Cytiva). After sample loading, the column was washed with 5 column volumes (CV) of wash buffer (100 mM Tris-HCl pH 8.0, 150 mM NaCl, 1 mM EDTA) to remove unbound material. Bound Strep-tagged Bdh2 was eluted isocratically with elution buffer composed of wash buffer supplemented with 50 mM biotin. Bdh2 elution was monitored by absorbance at 280 nm, and 0.5 ml fractions were collected. Fractions containing Bdh2 were identified by SDS-PAGE and pooled.

The pooled fractions were diluted threefold with 50 mM Tris-HCl pH 8.0 and subsequently loaded onto an anion exchange column (HiTrap Q HP, 5 ml; Cytiva) pre-equilibrated with the same buffer. Bound Bdh2 was eluted using a linear gradient from 0 to 1 M NaCl. Fractions containing pure Bdh2 were pooled, aliquoted, flash-frozen in liquid nitrogen and stored at −80°C.

### Metabolic cell extract preparation

Extraction and analysis of polar metabolites were performed as previously described in Malitsky et al. (2016) and Zheng et al. (2015) with some modifications: frozen yeast cell pellets were extracted with 1 ml of a pre-cooled (−20°C) homogenous methanol–methyl-tert-butyl-ether (MTBE) 1:3 (v/v) mixture, The tubes were vortexed and then sonicated for 30 min in ice-cold sonication bath (taken for a brief vortex every 10 min). Then, DDW–methanol (3:1, v/v) solution (0.5 ml) was added to the tubes, followed by centrifugation (15, 000 ***g*** for 10 min). The upper organic phase was discarded. The polar phase was re-extracted as described above, with 0.5 ml of MTBE, and the lower polar phase was transferred to a new Eppendorf, dried for 1.0 h by N_2_ to remove organic solvents, and then lyophilized.

### *Ex vivo* metabolomics assay

To investigate the enzymatic function of Bdh2, polar metabolites were extracted from *S. cerevisiae* ΔBdh2 cells grown to stationary phase (OD_600_≈16), harvested at 25 OD units per sample, and lyophilized. For the *in vitro* assay, samples were rehydrated with 400 μl of a reaction master mix containing 0.1 M sodium phosphate buffer (pH 8), 2 μM ZnCl₂, and 23 μM NADH. To initiate the reaction, 100 μl of purified Bdh2 protein (0.5 mg/ml, final concentration of 2 μM) or buffer control was added. Tubes were incubated at 30°C for 1 h without shaking, flash-frozen and submitted for untargeted metabolomics analysis.

### Image analysis

The images of the cells were detected, segmented and quantified by the software scanR Analysis (version 3.2; Evident). The total fluorescence intensity of each image is the median of the mean intensity per object in each well. The standard deviation of each image is the root of the sum of mean intensity per object in the well minus mean intensity per well, in the power of two, divided by the total number of objects per well. The intensity of GFP fluorescence (Figs 2B, 3) was calculated by deducting the mean intensity of all total intensities of the negative controls of the collection (non-GFP containing cells) from the total intensity of each well.

## Supplementary Material

10.1242/joces.264913_sup1 Supplementary information

Table S1.

Table S2.

Table S3.

Table S4.

Table S5.

Table S6.

Table S7.

Table S8.
