# Supplementary material for: A screening pipeline to characterize stress-induced enzymes uncovers a cellular function for the poorly characterized alcohol dehydrogenase Bdh2
Source: J Cell Sci. 2026 Jun 12;139(11):jcs264913. doi: 10.1242/jcs.264913 (PMC13286364; doi:10.1242/jcs.264913)
Supplement: Supplementary information [file joces-139-264913-s1.pdf]

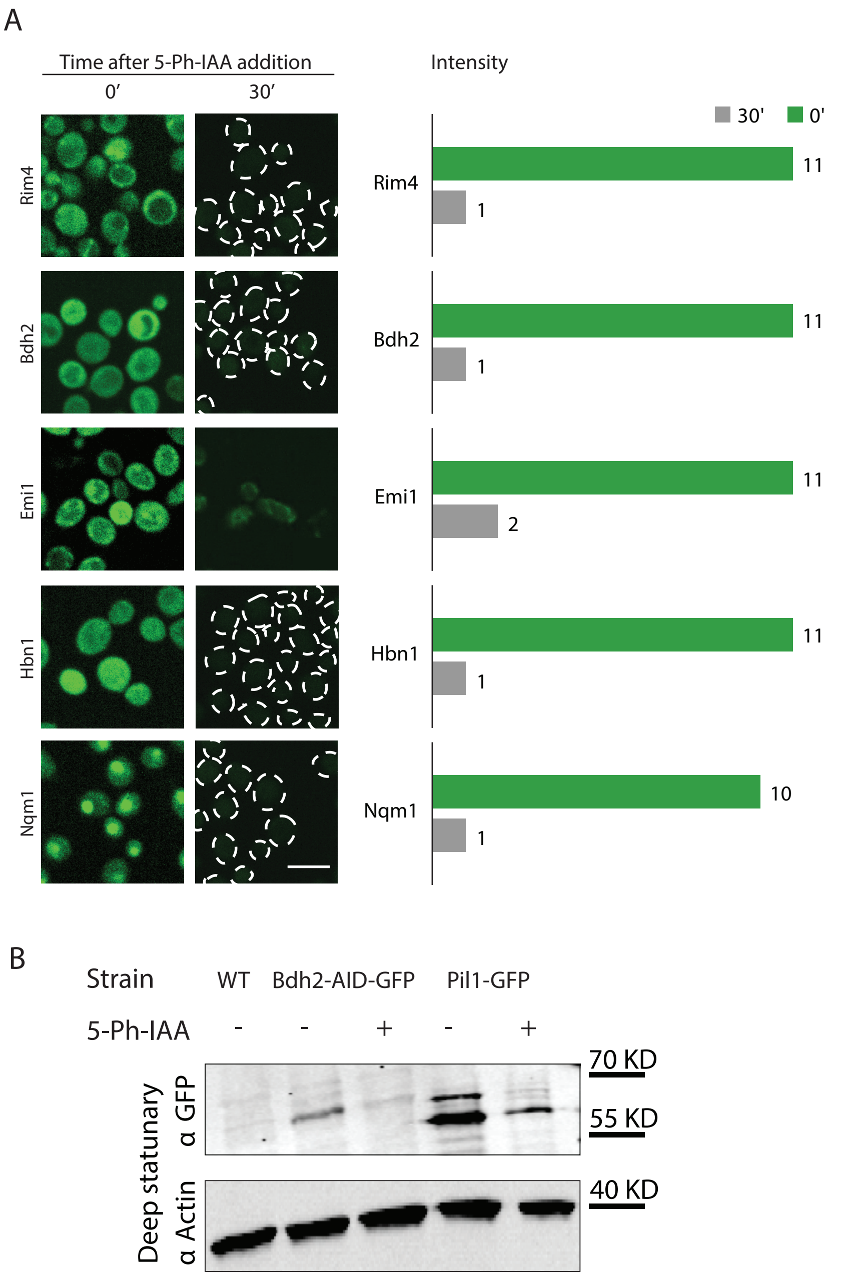

Fig. S1. A. Fluorescent images showing the strains with the targeted proteins that were responsive to 30 minutes (30') 5-Ph-IAA addition under the same condition in which they were expressed (left). Scale bar, 5 μm. The average fluorescent intensity of each strain, before and after depletion, is represented in the graph (right). B. A western blot showing the bands of Bdh2-GFP protein in control and Bdh2-GFP strains grown in deep stationary media, with and without the addition of 5-Ph-IAA. Actin levels were used as a control. A molecular weight ladder in Kilo Daltons (KD) is present on the right.

Journal of Cell Science • Supplementary information

Table S1.

Available for download at
https://journals.biologists.com/jcs/article-lookup/doi/10.1242/jcs.264913#supplementary-data

Table S2.

Available for download at
https://journals.biologists.com/jcs/article-lookup/doi/10.1242/jcs.264913#supplementary-data

Table S3.

Available for download at
https://journals.biologists.com/jcs/article-lookup/doi/10.1242/jcs.264913#supplementary-data

Table S4.

Available for download at
https://journals.biologists.com/jcs/article-lookup/doi/10.1242/jcs.264913#supplementary-data

Table S5.

Available for download at
https://journals.biologists.com/jcs/article-lookup/doi/10.1242/jcs.264913#supplementary-data

Journal of Cell Science • Supplementary information

Table S6.

Available for download at
https://journals.biologists.com/jcs/article-lookup/doi/10.1242/jcs.264913#supplementary-data

Table S7.

Available for download at
https://journals.biologists.com/jcs/article-lookup/doi/10.1242/jcs.264913#supplementary-data

Table S8.

Available for download at
https://journals.biologists.com/jcs/article-lookup/doi/10.1242/jcs.264913#supplementary-data

Journal of Cell Science • Supplementary information
